# MicroRNAs in fibrosis: opportunities and challenges

**DOI:** 10.1186/s13075-016-0929-x

**Published:** 2016-01-13

**Authors:** Steven O’Reilly

**Affiliations:** Faculty of Health and Life Sciences, Northumbria University, Ellison Place, Newcastle Upon Tyne, NE1 8ST UK

## Abstract

MicroRNAs (miRNAs) are small, non-coding RNAs that mediate mRNA cleavage, translational repression or mRNA destabilisation and are around 22–25 nucleotides in length via partial complementary binding to the 3′ untranslated region in target transcripts. They are master regulators of gene expression. Fibrosis is an important cause of morbidity and mortality in the world, and there are currently no accepted treatments for fibrosis. Many novel miRNAs are now associated with fibrosis, both organ-specific and systemic, as in the prototypical fibrotic disease systemic sclerosis. Recently, the targets of these altered miRNAs have been validated and defined new biochemical pathways. Dysregulated miRNAs are amenable to therapeutic modulation. This review will examine the role of miRNAs in fibrosis and the opportunities and challenges of targeting them.

## Background

Chronic inflammation often leads to tissue fibrosis [1]. Fibrosis is a wound-healing response that results from inflammation and results in excessive extracellular matrix (ECM) deposition that ultimately impairs the function of the tissue and organ. Fibrogenesis is choreographed by a variety of cytokines, chemokines and inflammatory cells. Various insults can cause tissue damage, including infectious agents, mechanical damage, heat and autoimmune disease. This stimulates a repair process that leads to repair of the tissue to restore homeostasis, and if not controlled appropriately this can lead to aberrant repair processes and ultimately fibrosis. In most cases, the injury is resolved and the fibrogenic response is controlled; however, if not, there is excessive ECM deposition and tissue remodelling and ultimately loss of function. In many diseases, fibrosis plays a major role, such as in liver or idiopathic lung fibrosis. In diseases such as systemic sclerosis (SSc), multiorgan fibrosis can lead to death [1] and these comprise a large proportion of deaths. Although a large number of diseases have a fibrotic pathology and represent a huge global burden, there are no currently licenced treatments. The paucity of therapeutic options likely reflects our lack of understanding of the underlying mechanism; however, in recent years, new targets have been discovered. MicroRNAs (miRNAs) are small (between 21 and 25 nucleotides long) non-coding RNAs that mediate mRNA repression or destabilisation and lead to translational repression and thus are important regulators of gene expression. MiRNAs initially were discovered in the nematode worm *Caenorhabditis elegans* but now are known to be highly conserved across species. They regulate gene expression post-transcriptionally and are both cell- and context-dependent. Recent evidence has suggested that miRNAs play a role in virtually all cell processes and that they also play a key role in many diseases, including fibrosis. It is now known that in the human genome it is composed of surprisingly few genes, and the complexity of gene expression is now recognised to be orchestrated by epigenetics. Over 60 % of all mRNAs are predicted targets of miRNAs [2] and thus their regulation in fibrosis development is significant. This review will examine the role of miRNA in fibrosis and the possibility of these being targeted therapeutically and the strategies to do so.

## MiRNAs

Unlike small interfering RNA (siRNA), miRNAs mediate their effects preferentially through imperfect base pairing with sequences in the 3′ untranslated region (UTR) of their target mRNAs. A region in the miRNA known as the seed region is important for repression of the target mRNA. The imperfect binding of the miRNA to the mRNA means that one miRNA can regulate many genes, and over 60 % of mRNAs are predicted to be targeted by miRNAs by computational prediction [2]. Many software programs publically available to determine targets of given miRNAs use this seed region to determine the possible cognate targets of miRNAs by conserved binding sites. Such databases include TargetScan (www.targetscan.org). Although these programs are useful, they are not absolutely correct and undoubtedly have false positives in there. There can be large differences in targets using different target prediction software. Furthermore, it has been found that miRNAs can target 5′ UTRs and it is now appreciated that they may actually be more promiscuous than first thought, underscoring their importance in post-transcriptional regulation. MiRs often target mRNAs that govern the same pathway that results in modulation of the pathway.

## Fibrosis signalling

The ultimate target cells in fibrosis are the fibroblasts where it produces a surfeit of ECM that ultimately impairs normal functioning of the tissue and organ. Inflammation is a key component of fibrosis and many cytokines and chemokines are involved in orchestrating this. Although there are many diverse proteins involved, transforming growth factor-beta (TGF-β) is considered the most important (see below). Other cytokines such as interleukin (IL)-6, IL-1, IL-13 and inflammasome components are also considered important mediators in disease pathogenesis. Also, molecules such as the neurotransmitter serotonin are important in mediating fibrosis but these may also lie upstream of TGF-β and converge on this molecule. Clearly, all these molecules may themselves regulate the expression of miRNAs involved in fibrosis. Table 1 demonstrates miRNAs in fibrosis and their confirmed targets.

**Table 1:** MicroRNAs in fibrosis

MicroRNA	Target gene	Pro- or anti-fibrotic	Tissue	References
17-5p	*Smad7*	Pro	Liver	[34]
21	Sprouty homolog-1, *PTEN*, *SMAD7*, *STAT3*, *PPAR-α*	Pro	Heart, skin, kidney	[22, 23, 63]
29, a,b,c	*Collagen1A1*, *1A2*, *4A5*, *FBN*, *ELN1*, *PDGFR*, *TAB1*, *ADAM*	Anti	Heart, skin, liver	[9, 14–17, 20]
33a	*PPAR-α*	Pro	Liver	[29]
122	*P4HA1*	Anti	Liver	[59]
129-5p	*Collagen1A1*	Anti	Skin	[39]
132	*MeCP2*	Anti	Liver	[41]
133a	*Collagens*	Anti	Liver	[28]
192	*ZEB1*	Pro	Kidney	[31]
199b	*DYRK1A*	Pro	Heart	[51]
199a-5p	*CAV1*	Pro	Lungs, skin	[47]
214	*CTGF*	Anti	Liver	[70]

## miRNA29 as a ‘master fibromiRNA’ regulator

The exact relative contribution of miRNAs to fibrosis and the bona fide targets are not fully known. MiRNAs can be either pro- or anti-fibrotic. However, one miRNA that has emerged as a master regulator appears to be miRNA-29. This is a family of miRNA29s and includes a, b, and c and differs in one, two, or three bases. These are clearly enmeshed in the pathogenesis of fibrosis. First described in fibrosis of the heart by van Rooij et al. in a landmark study, it has now been demonstrated to play a major role in fibrosis [3].

Decreased levels of miRNA-29 are associated with fibrosis in multiple organs, including the heart, liver [4], kidney [5] and skin, and in SSc [6], suggesting that this is a core ‘fibromiRNA’ [7]. In the heart, antagonism of miRNA-29 in vivo resulted in enhanced collagen in the tissue, whereas introduction of miRNA-29 mimics after heart damage reduced the collagen content [3]. Zhu et al. have also found downregulation of miRNA-29 in SSc fibroblasts and this correlated with the levels of its target gene collagen1A1 [8]. Whatever downregulates the miRNA-29a, this results in a de-repression of its targets that to date include the major structural collagens. We have found TAB1 to be a bone fide target of miRNA-29a in dermal fibroblasts [9]. This gene regulates TIMP-1, which blocks the action of matrix metalloproteinases, the enzymes that degrade ECM, and thus alters the balance of ECM turnover. Dysregulated TIMP-1 has been previously reported in SSc, and TIMP-1 is critical in hepatic fibrosis. Indeed, chemical inhibition of TAB1 also reduced ECM deposition [9], and we found downregulation of miRNA-29a in SSc fibroblasts [9]. MiRNA-29a has also been shown to be downregulated in SSc and is reduced by Toll-like receptor (TLR) 4 stimulation [10]. TAB is activated by TGF-β via the non-canonical pathway [11]. Furthermore, miRNA-29 has been shown to target platelet-derived growth factor receptor (PDGFR) [12], and platelet-derived growth factor signalling is known to be important in fibrosis [13]. Interestingly, the miRNA-29 family has been shown to target the DNA methyltransferases (DNMTs) and this results in methylation abnormalities [14]. Thus, reduced miRNA-29 could be targeting DNMTs, thus leading to hypermethylation and silencing of genes important in ECM regulation. Cushing et al. also showed that miRNA-29 regulates a variety of ECM genes that include the standard ECM proteins such as collagens and lamins but also integrins that themselves are involved in ECM regulation by activating latent TGF-β [15]. Furthermore, miRNA-29 is reduced in kidney fibrosis and is modulated by TGF-β, and the target of miRNA-29 was found to be disintegrin metalloprotease (a disintegrin and metalloproteinase, or ADAM) [16]. It was also found that miRNA-29 is reduced in lung fibrosis in both a TGF-β- and Smad3-dependent manner [17]. Smad3 genetically reduced mice were protected from fibrosis induced by bleomycin, confirming its regulation by TGF-β. Importantly, sleeping beauty transponson-mediated gene transfer of miRNA-29 to replace the reduced miRNA resulted in an attenuation of fibrosis [17]. This is an important step as this circumvents the need for the use of viral vectors that have problems associated with them, such as triggering host immune responses to the vector and labour costs. There is a further problem with the transduction, and the efficiency of viral vectors depends on the tropism of the virus for a particular tissue. A further study showed that diabetic kidney disease-associated inflammation and fibrosis are aggravated by genetic renal loss of miRNA-29b in mice, further underpinning the role of miRNA-29 in fibrosis. Gene replacement therapy of lost miRNA-29b expression in the mouse kidney reduced the fibrosis and inflammation in the diabetic kidney disease model, and the mechanism appears to include the TGF-β/Smad3 axis by modulating this [18]. Upregulation of miRNA-29a either by enforced overexpression or by the use of carveldilol, a beta-adrenoreceptor antagonist, was shown to reduce myocardial fibrosis mediated by experimental myocardial infarction in a small animal model [19]. Thus, agents that increase the levels of miRNA-29 would be predicted to reduce fibrosis. Although many cytokines that decrease the levels of miRNA-29 have been identified and these have been found to correlate in vivo, few, if any, cytokines have been found to increase the miRNA. In the bleomycin model of skin fibrosis, a mimic of SSc, it was found that miRNA-29a is supressed; however, restoration of miRNA-29a levels with the use of the tyrosine kinase inhibitor imatinib attenuated this fibrosis [6], suggesting that miRNA-29a is regulated by tyrosine kinase activation. It has recently been shown that miRNA-29a is also regulated by the alarmin IL-33 [20]. IL-33 is a danger-associated molecule that is released in tendon disease and this leads to downregulation of miRNA-29a and a subsequent increase in collagen levels. Recently, adenoviral overexpression of miR29a systemically injected ameliorated hepatic fibrosis induced by carbon tetrachloride [21].

## MiRNAs that modulate TGF-β signalling

Amongst the many cytokines fuelling fibrosis, TGF-β is one of the most pivotal cytokines mediating fibrosis in both organ-specific disease and SSc. TGF-β is critical in fibrosis but also in the development of the organism. It is elevated in many diseases and animal models of fibrosis and affects the activation of quiescent fibroblasts to ‘myofibroblasts’: the effector cells in fibrosis. The myofibroblast expresses high amounts of alpha-smooth muscle actin, secretes copious amounts of ECM and endows the cell with enhanced contractile force. TGF-β is secreted as a latent protein non-covalently linked to latency-activating protein (LAP) and through enzymatic proteolytic cleavage becomes ‘active’ after dissociating from LAP to bind to its cognate receptors. TGF-β then mediates gene expression via activation of the canonical Smad signalling pathway from transcriptional activation. MiRNAs that regulate TGF-β would therefore be of prime importance. Only recently have we begun to understand that miRNAs can be regulated by TGF-β and TGF-β itself can be regulated by miRNAs. One such miRNA is miRNA-21, which has been found to be upregulated by TGF-β stimulation. Furthermore, miRNA-21 is overexpressed in heart disease, and in a pressure overload model it was found that miRNA-21 overexpression modulates cardiac myocyte hypertrophy and interstitial fibrosis through reduction of sprouty homologue-1, a potent inhibitor of the extracellular regulated kinase/mitogen-activated protein kinase (ERK/MAPK) pathway, and thus elevates this signalling cascade [22]. In the bleomycin model of fibrosis, this increased miRNA-21 levels and the addition of antisense oligonucleotides to miRNA-21 reduced the severity of lung fibrosis even after a lag time of many days after lung injury [23]. Knockdown of miRNA-21 reduced pro-fibrotic responses of TGF-β stimulation, whereas increasing miRNA-21 enhanced the pro-fibrotic response [23]. It appears that the target mRNA of miRNA-21 is Smad7, which is an inhibitory Smad, attenuating the Smad-dependent signalling pathway. There is, of course, cross-talk between the Smad and other pathways, adding another layer of complexity. Smad3 itself regulates the expression of miRNA-21 by increasing this after stimulation with TGF-β; however, Smad2 inhibits this. Inhibition of miRNA-21 by ultrasound microbubble-mediated gene transfer of a negative miRNA-21 plasmid attenuated kidney fibrosis in an obstructive nephropathy model in mice, underscoring the importance of this miR [24]. MiRNA-21 has also been demonstrated to be elevated in SSc skin whole tissue and isolated fibroblasts [8]. Interestingly, miRNA-21 has a signal transducer and activator of transcription 3 (STAT3) binding site and this transcriptionally regulates the expression of miRNA-21, altering its targets [25]. STAT3 itself is an important molecule activated by IL-6 and mediates fibrosis in SSc fibroblasts [26]. A possible target of miRNA-21 is the gene phosphatase and tensin homolog (PTEN) and this abrogates a cell signalling pathway, resulting in increased ECM deposition [27]. It is known that the main substrate of PTEN is inositol-3,4,5-triphosphate and this activates Akt important in wound repair. It is also known that PTEN is reduced in SSc skin and that PTEN-ablated mice have exacerbated fibrosis compared with wild-type controls. Thus, elevated miRNA-21 reduces the expression of PTEN and this leads to altered Akt signalling, leading to increased ECM deposition. Interestingly, unsaturated fatty acids appear to upregulate miRNA-21 levels, inducing the suppression of PTEN in hepatocytes [27], suggesting a link between the metabolic syndrome, miRNAs and fibrosis.

TGF-β has been shown to downregulate miRNA-133a levels in hepatic stellate cells and during hepatic myofibroblast development without decreasing stimuli miRNA-133a levels [28]. Targets of miRNA-133a appear to be collagens, the major component of the fibrotic scar. Interestingly, serum expression of miRNA-133a was found to be elevated in patients with liver fibrosis and indicated the progression of liver fibrosis [28], indicating that this could be a valid biomarker where today none exists. For confirmation of liver fibrosis, often a biopsy is the only option but this, of course, is invasive. MiRNA-33a has also been shown to be modulated by TGF-β in stellate cells, and the potential targets of miRNA-33a are peroxisome proliferator activator receptor (PPAR) alpha and Akt in these cells. This is important as PPAR is critical in the shift from a quiescent to a myofibroblast in hepatic stellate cells [29].

MiRNA-192 is also upregulated by the addition of TGF-β1 and this upregulation promotes collagen deposition in a kidney fibrosis model [30]. Use of antagomirs to miRNA-192 reduced kidney fibrosis [30]. It appears that miRNA-192 targets the E-Box repressor Zinc finger E-box binding homeobox 1 (ZEB1) modulating E-cadherin levels, enhancing kidney fibrosis [31]. ZEB1 is a critical transcription factor in morphogenesis and epithelial-to-mesenchymal transition [32]. MiRNA-145 is also upregulated by TGF-β and this leads to increased fibrosis by increasing alpha-smooth muscle actin levels via activating latent TGF-β [33].

Recently, miR17-5p was found to be induced in hepatic stellate cells by TGF-β1 induction and activation and was also upregulated in the carbon tetrachloride model of liver fibrosis [34]. In vitro inhibition of miR17-5p led to reduction of cell activation and proliferation but no alteration in apoptosis levels [34].

## MiRNA-17-92 cluster

The miRNA-17-92 cluster which encodes seven miRNAs is important in oncogenesis (sometimes called OncomiRNA1), but this cluster also is important in fibrosis. It is critical in epithelial lung development, and mice that lack this cluster do not have many lung epithelial cells and die shortly after birth. Mice in which transgenic overexpression in the lungs of this miRNA cluster results in lung hyperproliferation and blocks differentiation of lung progenitor cells were also demonstrated with transgenic technology [35]. It was found in idiopathic pulmonary fibrosis samples that miRNA-17-92 cluster was reduced and this was confirmed by both polymerase chain reaction and in situ hybridisation techniques. It was found that there was hypermethylation of DNA in the promoter region of miRNA-17-92 and that this was leading to repressed expression of the cluster; the enzyme DMNT-1 is altered in pulmonary fibrosis and this is also a target gene of some of the miRNAs in the cluster. Thus, a complicated feed-forward loop exists in which miRNAs from the gene cluster target DNMT-1; thus, the reduction of the miRNAs leads to enhanced DNMT-1 levels. Transfection of miRNAs reduced DNMT-1 levels and thus globally methylated DNA levels. Also, in vitro and in vivo administration of 5′aza′2-deoxycytidine, a global demethylation agent, restores the reduction of the miRNA cluster and reductions in their targets such as collagen1A1 and CTGF. In vivo treatment of bleomycin-treated mice after commencement of fibrosis attenuated the fibrosis in the lungs and also elevated levels of the miRNA cluster under examination. This was accompanied by a reduction in DNMT-1 enzyme levels as this is targeted by the miRNA and also a reduction in pro-fibrotic genes driving the fibrosis [36]. This cluster has been implicated in liver fibrosis with the bona fide target identified as CTGF mediated via p53, a tumour-related gene [37]. CTGF is important as a fibrotic molecule in its own right and is often associated with TGF-β.

## MiRNA-129-5p

In the prototypic fibrotic disease SSc, there is an increase in leucocytes in the skin, including primarily T cells. These T cells that are residing in the skin are in close proximity to the myofibroblasts, suggesting that they are governing their transdifferentiation [38] and may activate other immune cells in the inflammatory foci. It has been described in SSc fibroblasts that miRNA-129-5p is repressed compared with healthy control fibroblasts [39]. They also show that the T-cell cytokine IL-17 can increase miRNA-129-5p levels, and using siRNA to knock down IL-17 receptors in dermal fibroblasts reduced miRNA-129-5p levels. The actual target mRNA of miRNA-129-5p appears to be collagen alpha-1 [39]. This all suggests that the Th17 cells reduce collagen expression via the upregulation of the negative regulator miRNA-129-5p; however, in SSc fibroblasts, the dysregulated TGF-β levels alter the cells’ sensitivity to IL-17, possibly through downregulation of the TGF-β receptors, thus leading to reduced IL-17 stimulation and a decrease in miRNA-129-5p and ultimately leading to enhanced collagen levels. This hypothesis requires further investigation but may explain the inconsistent reports on whether IL-17 is pro- or anti-fibrotic [40]. Increasing knowledge on T cells and their factors mediating perturbations in miRNAs will help elucidate these interactions.

## MiRNA-132

**Fig. 1:**
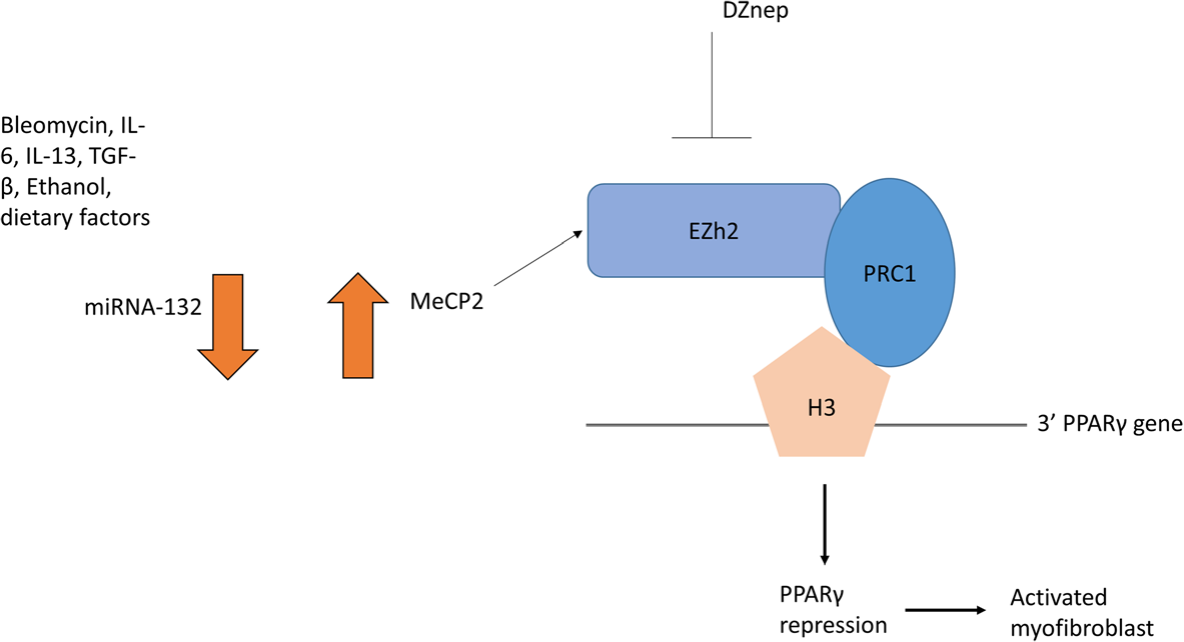
miRNA-132 feed-forward loop. Various factors such as IL-6 and dietary factors, including alcohol, reduce the expression of miRNA-132 and thus lead to increases in its target mRNA meCP2, and meCP2 interacts with the methylase Ezh2 that methylates lysine27 on H3 and interacts with PRC1; this all leads to repression of the master regulator PPARγ and myofibroblast activation and extracellular matrix expression. Blockade of Ezh2 with DZnep may be beneficial in fibrosis through blocking histone methylation. *Ezh2* enhancer of Zeste homologue 2, *IL* interleukin, *MeCP2* methyl cap-binding protein 2, *miRNA* microRNA, *PPARγ* peroxisome proliferator activator receptor-gamma, *PRC1* polycomb recessive complex 1, *TGF-β* transforming growth factor-beta

It was found that miRNA-132 regulates the expression of the methyl cap-binding protein 2 (MeCP2) and that transdifferentiation of liver fibroblasts into the ‘myofibroblast’ of the liver stellate cells is associated with huge suppression of miRNA-132 [41], suggesting that negative regulation of MeCP2 is controlled by miRNA-132 expression. In liver fibrosis models such as the carbon tetrachloride model, miRNA-132 was reduced, leading to an upregulation of its target MecP2; this in turn regulates the expression of the methyl enzyme enhancer of Zeste homologue 2 (Ezh2). This enzyme is a methyltransferase which methylates histone H3 at lysine 27 and affects transcription of genes. Methylation is associated with repression of gene expression. This all culminates in a suppression of PPAR-γ and the brake being ‘released’ on ECM genes [41] (Fig. 1). This is an example of a complex interplay between all three epigenetic mechanisms working in a feed-forward loop and illustrates the cross-talk between epigenetic modifications. Epigenetic regulation of such processes is complicated and only now is beginning to be understood. Because epigenetic modifications are malleable and modulated by the environment, they can be modulated by hormones and diet.

## MiRNA-155

Elevated expression of miR-155 has been demonstrated in alcoholic liver disease in both humans and mice [42]. It is now known that miR-155 is a critical regulator of the inflammatory response, especially that of the pattern recognition receptors, the TLRs [43], and is a negative regulator to limit the response. One of the major ligands for TLR4 is lipopolysaccharide (LPS) and this has been found to alter the expression levels of miR-155 [43]. LPS has been proposed to be a trigger of liver fibrosis through enhanced translocation of bacteria from a ‘leaky’ gut due to alterations in the gut permeability as a consequence of alcohol provoking an inflammatory and fibrotic response [44]. In diet-induced obesity models of liver disease in which the obesity leads to fatty and fibrotic livers, miR-155 is induced, but under basal conditions the miR-155 expression profile is low. In mice with miR-155 deleted genetically, these are more susceptible to diet-induced fatty liver disease as compared with wild-type mice [45] and the target of this is the liver X receptor [45]. The liver X receptor modulates lipid deposition genes and the hypothesis is that miR-155 upregulation is a protective mechanism due to targeting the liver X receptor and thereby supressing its expression and downstream genes. It was recently demonstrated in liver disease that miR-155 is elevated, confirming previous reports, and that this is altered in stellate cells [46]. Importantly, the miR alters ERK and inhibiting miR-155 stimulates the fibrotic response.

## MiRNA-199a

Very recently, miRNA-199a-5p was also found to be upregulated in lung fibrosis. Interestingly, the elevated miRNA-199a-5p was localised in myofibroblasts and not adjacent non-myofibroblast cells [47]. In a quest to identify targets, the authors used a gene array approach and found that caveolin-1 was a target and this was confirmed by using luciferase assays that had mutated binding sites, confirming this as a true target of miRNA-199a-5p [47]. Caveolin-1 is itself a target of the pro-fibrotic cytokine TGF-β, and in three different models of different organ fibrosis, it turns out that miRNA-199a-5p is commonly dysregulated. This suggests that this miRNA is a core regulator of fibrosis. It is of interest that caveolin-1 is a target of miRNA-199a-5p because caveolin-1 is part of caveolae, which are bulb-shaped invaginations on the cell membrane, around 50–100 nm, and are involved in cell signalling and trafficking. Caveolin-1 mediates TGF-β signalling by internalising the TGF receptors into caveolae and this internalisation by these buds leads to degradation and blunting of the signal and thus less Smad activation. It was found that Caveolin-1 protein expression is reduced in the skin and lungs of patients with SSc, and reconstitution of the reduced caveolin-1 reduced the expression of key ECM [48]. Single-nucleotide polymorphisms have also been found to be associated with caveolin-1 and fibrosis [49]. Reduced caveolin-1 is associated with idiopathic pulmonary fibrosis and this enhanced TGF-β signalling through modulation of the MAPK pathway [50]. There is now clear evidence that miRNA-199 is involved in fibrosis and is a therapeutic target, and further fibrosis animal models will glean more information on clinical utility.

## MiRNA-199b

Recent studies revealed that miRNA-199b is an important miRNA in mediating fibrosis. It was shown to be elevated in heart disease and in hypertrophy models on heart disease. It targets dual-specificity tyrosine (Y) phosphorylation-regulated kinase-1a (dyrk1a), which alters calcenurin levels and modulates the signalling through NFAT. Genetically modified mice that overexpress miRNA-199b have enhanced myocardial fibrosis, and in vivo use of an antagomir against miRNA-199b in vivo reduced myocardial fibrosis in multiple models of heart disease [51].

## Modulation of miRNAs for therapeutic gain

**Fig. 2:**
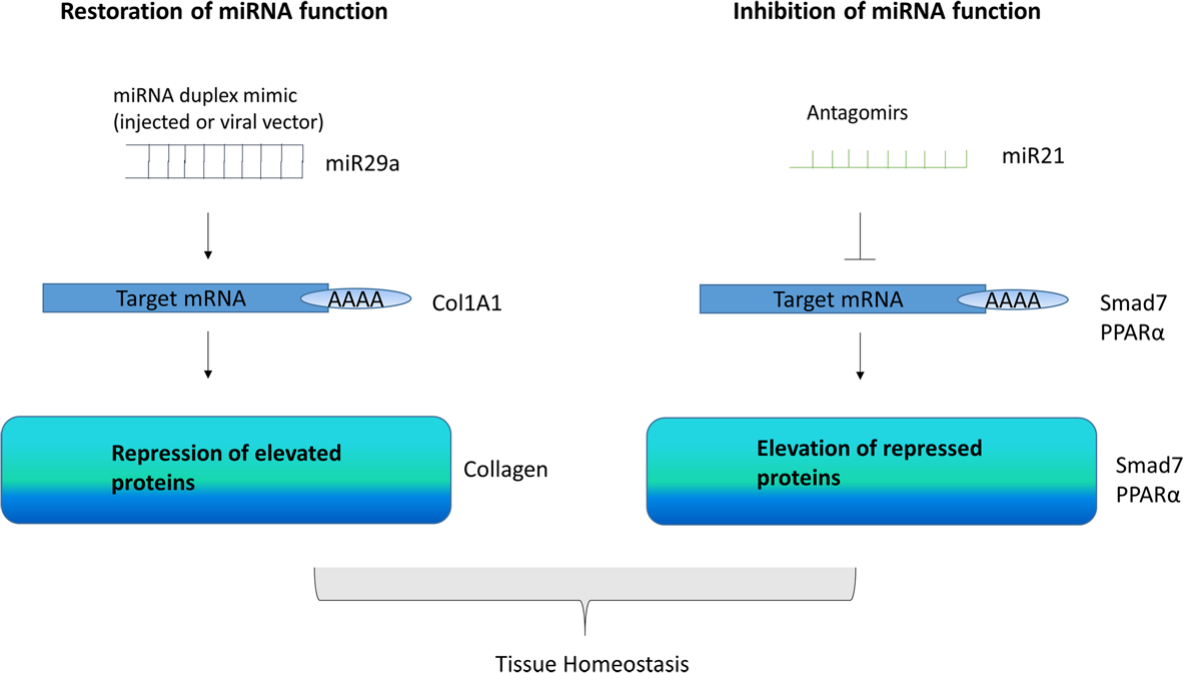
miRNA modulation. Two methods are employed for miRNA therapeutics restoration of miRNA or inhibition. Restoring miRNA is through the use of double-stranded RNA, which is composed of a guide and passenger strand that is chemically modified usually by a cholesterol modification. The guide strand is identical to the miRNA that is diminished. This is then incorporated into the RISC complex and the target mRNAs reduced. Inhibition of miRNA function is achieved through single-stranded chemically modified LNA antagomirs which can be cholesterol-conjugated and have increased stability. These molecules bind the mature miRNA and stop them from being loaded into the RISC, therefore increasing the mRNA target(s). In the context of fibrosis, to the right of the illustrations are examples of miRNA as both a mimic and antagomir binding the target mRNA and altering the protein output. Smad7 is a negative regulator of the fibrotic cascade, so elevated levels reduce fibrosis. PPAR-α is also a negative regulator of fibrosis and thus its restoration is positive. *miRNA*, MicroRNA, *PPARα* peroxisome proliferator activator receptor-alpha, *RISC* RNA-induced silencing complex

Genetic ablation studies of specific miRNAs in animal models and also in vitro activation or inhibition have established miRNAs as an attractive target for therapeutic modulation in multiple diseases [52] (Fig. 2). MiRs are appealing therapeutically since multiple similar pathways can be targeted at once because each miRNA targets multiple mRNAs and the molecules are small and easily chemically modified. Also, miRs are often conserved across species and so the same oligos can be used in animal models. Chemical modifications to the miRNAs include locked nucleic acid (LNA) technology in which the ring in the sugar phosphate backbone is chemically locked by the introduction of a 2′-O,4′-C-methylene bridge. This increases the stability of the molecule and resistance to breakdown via endogenous nucleases. RNA itself is rather unstable. LNA technology modification increases the RNA melting temperature by 2.4 °C per LNA monomer introduced and increases the binding affinity of the nucleic acid to the mRNA [53]. LNA-modified antagomirs were first used in vivo in non-human primates to target the liver miRNA-122 and resulted in a sustained and long-lasting decrease in cholesterol [54]. Injection of unmodified miRNAs into the tail vein of mice results in breakdown and renal excretion very quickly [55]. A different chemical modification is a 2′O-methyl modification that increases nuclease resistance. Another chemical modification is the addition of a cholesterol moiety at the 3′ end. Despite the theoretical rationale for modulation, only one antagomir is currently in clinical use. This is the antagomir for miRNA-122, marketed as Miravirsen (Roche, Copenhagen, Denmark), and has shown great clinical efficacy [56]. Miravirsen targets miRNA-122, which is a liver-specific miRNA and thus is not expressed anywhere else; it uses LNA technology. It works by blocking the interaction with the miRNA (122) and its target RNA in the hepatitis C virus (HCV) 5′ UTR [57]. MiRNA-122 is essential for the replication of HCV [58] and lipid and iron metabolism in the liver [59]. It appears safe and well tolerated and has no adverse effects. Another approach to targeting miRNAs is termed tiny LNAs. These are tiny (8-mer) LNA anti-miRNAs that target only the conserved seed region [60]. An alternative strategy is the use of miRNA masking (miRNA-Mask); this works by the introduction of single-stranded RNA that targets the 3′ UTR of the protein coding mRNA and thus ‘covers up’ the binding site to the miRNA and thereby de-represses the protein target. This technology has been used to confirm targets of miRNAs in vitro. Further study is needed to determine which chemical modification is optimum for stability without undue toxicity. Another way of targeting miRNAs in fibrosis is to reconstitute reduced levels of the miRNA with miRNA replacement, thus restoring the suppression of the putative target(s). The introduction of such miRNA mimic replacement can occur through the direct introduction of the mimic or the use of viral vectors to express the miRNA. Viral vectors, however, may induce an unwanted immune response, and tissue tropism may limit their effectiveness. Therapeutically, antagomirs are much more advanced than miRNA replacement therapy. One issue that is currently hampering the field is that the introduction of miRNA therapeutics is not cell- or organ-specific and thus being systemic may have major side effects. Getting the miRNA therapeutic to the correct target tissue remains a challenge, especially if injected systemically; getting the miRNA out of the circulatory system and crossing the endothelium will be challenging if over 5 nm in diameter. One recent study used a neutral lipid emulsion as a vehicle for the systemic delivery of miRNA-34a, which is downregulated in cancer, and this lipid vehicle delivery system was capable of reducing lung tumour burden in a mouse model of lung cancer [61]. It is suggested that the use of a neutral lipid vehicle does not aggregate so much and is less likely to be engulfed by macrophages [61]. As further delivery systems are developed for miRNAs to target them more specifically to the target tissue and cells, further widespread use will occur. A further issue with the blockade of miRNAs with antagomirs may be that the levels of redundancy may restrict the therapeutic benefit.

The vista is optimistic for using miRNAs therapeutically to treat fibrotic conditions such as SSc. This is one of the fastest growing areas in scientific research and offers the potential to revolutionise treatment. It was shown in an animal model of kidney fibrosis that the use of an antagomir to miRNA-214 attenuated kidney fibrosis [62] and that this is independent of classic TGF-β signalling.

One of the most studied fibrotic miRNAs is miRNA-21, and use of an antagomir in two mouse models of kidney fibrosis demonstrated great clinical efficacy in reducing fibrosis [63]. Mechanistically, it was found that miRNA-21 targets the 3′ UTR of PPAR-α, an important receptor in involved in lipid metabolism; indeed, deletion of PPAR-α abrogated the effect of anti-miRNA-21 anti-fibrotic effects [63]. The authors further demonstrated that mitochondrial oxidative regulators are also a target of miRNA-21 [63], suggesting that metabolic stress is important in fibrosis generation. Regulus Therapeutics (San Diego, CA, USA) also has an antagomir against miRNA-21 (RG-012) in the developmental pipeline and this antagomir showed good efficacy in animal models of Alport syndrome, an inherited kidney disease in which mutations in collagen lead to excess collagen deposition within the kidney.

## Replacing reduced miRNAs

Replacement of reduced miRNA expression by using miRNA mimics could be one therapeutic strategy in fibrosis. This involves the use of RNA duplexes in which there is a ‘guide strand’ and a passenger strand that may be chemically modified. Although restitution of miRNAs is in its infancy, it has been proven in animal models of cancer and more recently fibrosis. Often restoring the lost miRNA is achieved by viral vectors. This has its own challenges, as the vector size may be large and systemic delivery may be difficult to achieve and can also have high immunogenicity. Montgomery et al. recently published the use of a miRNA-29 mimic in an animal model of fibrosis, the bleomycin lung fibrosis model, and showed that introduction of the miRNA mimic in vivo was effective in reducing this fibrosis [64] and confirmed reductions in the miRNAs target proteins. But what is more impressive is that introduction of the mimic was effective in prevention of fibrosis with no observable side effects [64]. miRagen Therapeutics (Boulder, CO, USA) has an miRNA-29 mimic in pre-clinical development for fibrotic conditions and uses a non-viral vector for systemic delivery. miRagen Therapeutics should move into clinical trials soon with this molecule. (Table 2 illustrates miRNA therapeutics in development.)

**Table 2:** MicroRNA therapeutics in fibrosis

Company	MicroRNA target	Stage
Regulus Therapeutics	21 (RG-012)/Antagomir	Phase 1 clinical trial Alport syndrome
miRagen Therapeutics	29 mimic	Pre-clinical development
miRagen Therapeutics	155^a^ mimic	Pre-clinical
Marina Biotech	21 Antagomir	Pre-clinical

^a^Although developed for immune modulation could be useful in fibrosis

Although miR therapeutics is a promising area, challenges do remain. These include the effective delivery of the mimic or antagomirs targeted to the correct tissue and cell type. Strategies to do so include viral vector delivery, liposomes and nanoparticle delivery. Of course, some tissues such as the skin and lung are more accessible than others. The use of ligands that are attached to the nucleotides that are specific for certain cell surface receptors that bind and then internalise may be one way of enhancing specific cell uptake. Here, they can release their ‘cargo’. A major concern is also ‘off target effects’ of the miR therapeutics. A principle benefit of targeting miRNAs is that they often target mRNAs in the same pathway and so fine-tune the output of a particular pathway; however, this may be a wanted effect in one specific cell type but not in another cell type. A further consideration should be drug resistance, as in cancer treatment drug resistance can develop; this itself can be mediated through miRs, altering gene expression of mRNAs critical in driving this effect. It could be speculated that, in miR therapeutics, antagomir therapy may reduce the miR and thus increase its targets, but mechanisms to counter this through upregulation of the miR being targeted by increased biogenesis may lead to diminishing effectiveness. Finally, the TLR system can also respond to RNA and induce an antiviral response through downstream adaptor proteins and this could be theoretically activated by miRs.

## Serum miRNAs as diagnostic/prognostic markers

MiRNAs appear remarkably stable in serum and other bodily fluids such as urine and saliva [65]; this is because they are enclosed in extracellular membrane-bound vesicles or combined with high-density lipoproteins. Thus, they are attractive as a non-invasive diagnostic biomarker of disease. There are associations with various cytokines in fibrotic diseases; however, none of these is sufficiently robust to be diagnostic or guide treatment. The hepatic-specific miRNA-122, which is the target of miravirsen, has been shown to be reduced in hepatic fibrosis in cells [66]; however, the circulating serum levels of miRNA-122 are elevated significantly in fibrosis and this correlates with serum alanine aminotransferase (ALT) levels, a marker of damaged liver [67] and may be a more reliable marker than ALT in assessing fibrosis [67] and has been found in other independent cohorts [68]. It has been suggested that serum miRNA-122 is elevated in hepatocyte injury regardless of the aetiology [69] and this may prevent it being used specifically for liver fibrosis. miRNA-214 has also been found extracellularly and to actually mediate signals through downregulation of its target gene CTGF via extracellular exosomes to target cells mediating fibrosis [70]. This has also been shown in in vitro models and in vivo [71]. It now appears that exosome mediates the transfer of miRNAs to distal cells to trigger intracellular signalling and that exosomes display cell surface markers that the target can recognise and internalise through receptors, thereby giving rise to direct mRNA repression. The important fibrotic miRNA-29a has also been found in the serum to be associated with cardiac fibrosis in cardiac hypertrophy [72]. In SSc, a few circulating miRNAs have been demonstrated to be higher in serum compared with controls, including miRNA-142-3p [73]. Furthermore, miRNA-196a has been demonstrated to be reduced in SSc serum compared with healthy controls [74]. There was also a correlation between lower serum miRNA-196a levels and the modified Rodnan skin score, which is a measurement of skin thickness due to fibrosis [74]. Indeed, levels of miRNA-196a have also been found to be differentially expressed in the hair of patients with SSc [75]. Although miRNAs in serum have been demonstrated in association with fibrotic organ-specific diseases or in SSc, their clinical utility remains to be determined, and larger studies are needed to validate these reports. Stratification of patients on the basis of specific miRNAs may allow a more targeted therapeutic approach. Furthermore, in the case of serum miR, there is no consensus yet on the ‘normalisation’ method used for these studies. Tissue-based markers of miR expression are of limited value as whole tissue comprises multiple cell types and miRs are cell-type dependent. The additional effort to isolate single cells yields much more informative results.

## Conclusions

Since their discovery two decades ago, miRNAs have been found to be associated with various diseases. There is currently good evidence for the role of miRNAs in fibrotic diseases, either organ-specific or systemic fibrosis, such as SSc. Important steps have been made in recent years, including the identification of dysregulated miRNAs and their targets. Whereas the exact targets of these miRNAs are unknown for some, they are known for others, and they are regulating key downstream pathways in disease pathogenesis such as miRNA-29, which is a key mediator of fibrosis. Gene therapy with the restoration of miRNA-29, at least in an animal model of fibrosis, appears to reduce fibrosis [18]. Currently, many companies are looking at using miRNA technologies for modulating various fibrotic conditions. By far the most advanced is antagomirs to block the function of an miRNA and thus increase its target transcript and signalling pathway. However, recent data are emerging on the role of increasing miRNAs in vivo, especially miRNA-29a, to restore its function and thus suppress fibrosis without the need for viral vectors. There are some problems, such as stability of the miRNAs and tissue targeting, but if these are overcome, therapeutic modulation of miRNAs may become a clinical reality in fibrosing conditions, which is a huge unmet clinical need. The use of vehicles to target specific tissues and cells is important to direct where the target should be modulated, and optimisation of such vehicles is critical. Inherent to understanding the manipulation of miRNAs is a deeper understanding of the targets of miRNAs and the regulation of their expression in vivo. Caution must be taken when interpreting the expression of miRNAs in tissues as miRNAs are cell type-specific and the value of whole tissue expression such as skin may be questionable. Techniques such as laser capture micro-dissection in which individual cells can be captured are more useful in deriving meaningful results of miRNA analysis. In heterogeneous diseases such as SSc or liver fibrosis, it may be that the clinical course of the disease is dictated by the expression of miRNAs, which themselves are plastic and altered by the environment.

## Abbreviations

ALT: Alanine aminotransferase
DNMT: DNA methyltransferase
ECM: Extracellular matrix
ERK: Extracellular regulated kinase
HCV: Hepatitis C virus
IL: Interleukin
LAP: Latency-activating protein
LNA: Locked nucleic acid
LPS: Lipopolysaccharide
MAPK: Mitogen-activated protein kinase
MeCP2: Methyl cap-binding protein 2
miRNA: MicroRNA
PPAR: Peroxisome proliferator activator receptor
PTEN: Phosphatase and tensin homolog
siRNA: Small interfering RNA
SSc: Systemic sclerosis
STAT3: Signal transducer and activator of transcription 3
TGF-β: Transforming growth factor-beta
TLR: Toll-like receptor
UTR: Untranslated region
ZEB1: Zinc finger E-box binding homeobox 1
